# Role of hepatic stellate cells in liver ischemia-reperfusion injury

**DOI:** 10.3389/fimmu.2022.891868

**Published:** 2022-07-28

**Authors:** Yuming Peng, Qiang Yin, Miaoxian Yuan, Lijian Chen, Xinyi Shen, Weixin Xie, Jinqiao Liu

**Affiliations:** ^1^ First Department of General Surgery, Hunan Children’s Hospital, Changsha, China; ^2^ Zhaolong Chen Academician Workstation, Changsha, China; ^3^ Department of Ultrasound, Hunan Children’s Hospital, Changsha, China

**Keywords:** ischemia-reperfusion injury, hepatic stellate cells, hepatocytes, Kupffer cells, neutrophils, sinusoidal endothelial cells, liver transplantation, liver

## Abstract

Liver ischemia-reperfusion injury (IRI) is a major complication of liver trauma, resection, and transplantation. IRI may lead to liver dysfunction and failure, but effective approach to address it is still lacking. To better understand the cellular and molecular mechanisms of liver IRI, functional roles of numerous cell types, including hepatocytes, Kupffer cells, neutrophils, and sinusoidal endothelial cells, have been intensively studied. In contrast, hepatic stellate cells (HSCs), which are well recognized by their essential functions in facilitating liver protection and repair, have gained less attention in their role in IRI. This review provides a comprehensive summary of the effects of HSCs on the injury stage of liver IRI and their associated molecular mechanisms. In addition, we discuss the regulation of liver repair and regeneration after IRI by HSCs. Finally, we highlight unanswered questions and future avenues of research regarding contributions of HSCs to IRI in the liver.

## Introduction

Liver diseases have become one of the leading causes of death worldwide in the past few decades. It was estimated that over two million deaths were attributed to major liver diseases, such as cirrhosis and liver cancer (1), highlighting the demand for liver transplantation, which is currently the optimal treatment for end-stage liver diseases. Upon restoration of blood supply after interruption, the liver subjects to further injury that aggravates the initial injury caused by ischemia. This pathophysiological process is called liver ischemia-reperfusion injury (IRI) (2). Liver IRI can be classified into warm and cold IRI, which share similar mechanisms with differences mainly in the clinical settings (3). Warm IRI, initiated by hepatocellular damage, develops *in situ* during liver trauma and transplantation where hepatic blood flow falls transiently. Cold IRI, initiated by liver sinusoidal endothelial cells (LSECs) damage and microcirculatory disruption, occurs ex vivo during cold storage of the liver before transplantation surgery (4). Liver IRI is a critical complication in several clinical settings including liver trauma, resection, and transplantation (5–7). The degree of liver IRI depends on the period, methods of ischemia, and baseline liver condition (8). Continuous occlusion as short as 5 min can still lead to liver damage 1 d postoperatively in rat model of liver IRI, and IRI is much exacerbated in steatotic liver (9). IRI is an important cause of liver dysfunction (10), yet no reliable treatment option has been discovered. A better understanding of the cellular and molecular mechanisms of liver IRI may lead to improvements to clinical outcomes of liver disease patients, particularly those undergoing liver transplantation.

Substantial knowledge has been accumulated in regard to mechanisms underlying hepatic ischemic injury due to success of animal models. It is proposed that liver IRI consists of initial and late phases with distinct pathophysiological characteristics. Initial phase of liver IRI occurs 1-3 h after reperfusion (11–13), and manifests as rapid Kupffer cell activation after reperfusion (11, 14). Reactive oxygen species (ROS) is released by Kupffer cells, leading to oxidative stress and liver injury. Subsequently, the early liver injury triggers the release of a series of pro-inflammatory cytokines, such as TNF-α and IL-1β, inducing immune cell recruitment and more severe liver injury (15). The late phase of liver IRI, which occurs at 6-24 h after reperfusion (11, 12), is characterized by the recruitment of neutrophils to the liver and subsequent damage to hepatocytes (14).

Multiple cell types, including hepatocytes, liver sinusoidal endothelial cells (LSECs), Kupffer cells, hepatic stellate cells (HSCs) extrahepatic macrophages, neutrophils, and platelets, are involved in the progression of liver IRI (14, 16). Hepatocytes and LSECs are the main cell types subject to IRI induced cell death (17). Extensive studies indicate that Kupffer cells play a critical role in regulating IRI by promoting inflammatory injury mediated by cytokines and chemokines (2, 17). Neutrophils act as the main actor of cell injury during liver IRI following their recruitment to the liver regulated by Kupffer cells releasing of chemokines. Upon migration and infiltration to the liver, neutrophils respond to signals released by injured hepatocytes, inducing release of ROS and degranulation to cause further injury (17, 18). HSCs, which reside in the perisinusoidal space of liver and are known for their essential function of regulating hepatic fibrosis (19), has not been long investigated in liver IRI. As more recent studies shed light in the role of HSCs in liver IRI, we aimed to summarize the effects of HSCs on regulation of liver IRI in both injury and repair/regeneration stages, their intercellular communications with other cell types during IRI, and the associated molecular mechanisms.

## Quiescence and activation of HSCs in liver IRI

HSCs are localized in the subendothelial space of Disse between hepatocytes and LSECs and comprise approximately 15% of total cells in human liver (20). Due to anatomic features, intercellular crosstalk can occur between adjacent cell types including hepatocytes, Kupffer cells, bone marrow-derived macrophages, LSECs, infiltrating leukocytes, and nerve cells (20, 21). HSCs are identified by expression of both mesenchymal and neuronal markers including desmin, vimentin, nestin, and glial fibrillary acidic protein (GFAP) (22). Under physiological circumstance, HSCs sustain a non-proliferative and quiescent phenotype with angular, rounded cell bodies, extended cytoplasmic processes, and unique vitamin A storage in lipid droplets (23). In normal liver, HSCs contribute to liver regeneration, regulation of sinusoidal circulation, and vitamin A storage and release (24–26). HSCs are important sources of myofibroblasts during liver damage (27). When liver injury occurs, however, HSCs become activated and transdifferentiate into proliferative, contractile, and inflammatory myofibroblasts, which are characterized by secretion of extracellular matrix (ECM) molecules (28, 29). In this condition, HSCs are marked by expression of α-smooth muscle actin (α-SMA) (30). Activated HSCs secrete endothelin-1 (ET-1), which is a molecule with potent vesoconstricting effect, promoting proliferation and fibrogenesis, and thus is supposed to contribute to portal hypertension (31, 32). HSCs have been identified as a critical driver of fibrosis in liver injury (19, 33). It is postulated that during liver IRI, HSCs are activated by TNF-α, IL-6, and nitric oxide (NO), followed by transdifferentiation into myofibroblast phenotype (17). Activation of HSCs results in secretion of matrix metalloproteinases (MMPs), cytokines, and chemokines, leading to ECM destruction, further activation of HSC, and infiltration of neutrophils and platelets (17). These effects imply HSCs can play an important role in regulating hepatic inflammation during IRI.

## Effects of HSCs on liver IRI

**Figure 1:**
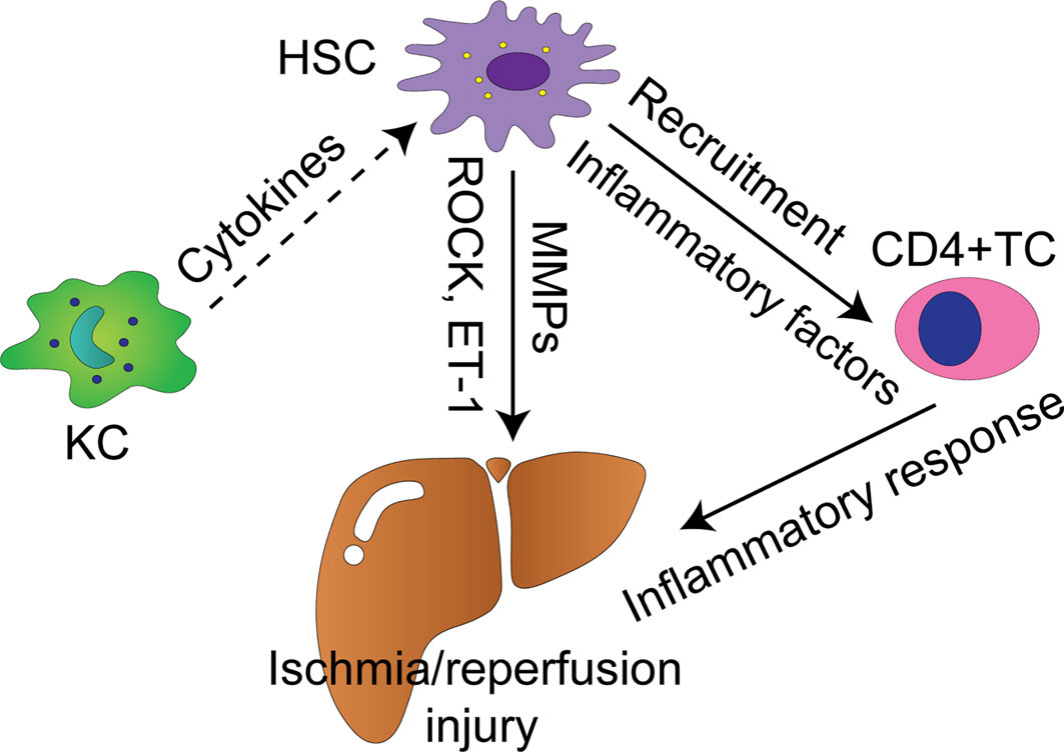
Cellular and molecular mechanisms by which HSCs modulate liver IRI. Solid arrows indicate positively regulatory effect with supporting experiment data, whereas dash arrows indicate putative positively regulatory effect. KC, Kupffer cell; TC, T cell; HSC, hepatic stellate cell; MMPs, matrix metalloproteinases; ROCK, Rho-associated coiled-coil forming protein serine/threonine kinase; ET-1, endothelin-1.

Functional roles of HSCs in injury stage of hepatic IRI has received much less attention than Kupffer cells, partly because functional inhibitor of Kupffer cells, gadolinium chloride, has enabled direct manipulation of these cells in experimental models (34). HSCs as a whole promote liver damage in the early phase of IRI, but they may mediate protective effect upon some pharmacological interventions or external stimuli as well. Thus HSCs may be regulated by specific signaling to combat IRI in the liver. **Figure 1** summarizes the molecular mechanisms of liver IRI mediated by HSCs, which are discussed below.

The involvement of HSCs in liver IRI was proposed based on preliminary observational studies. For instance, Takeda et al. found that heparin diminished serum levels of ET-1, aspartate transaminase (AST) and recovered hepatic IRI induced disturbance of oxidized and deoxidized hemoglobin after 1 h of IRI in rabbit model. Interestingly, electron microscopy revealed that IRI induced structural alteration of HSCs, which were target cells for ET-1, was normalized upon administration of heparin (35). These results suggested that HSCs mediated liver protective effect of heparin *via* microcirculation regulatory molecule ET-1. Further studies supported HSC’s role in hepatic IRI was partly mediated by regulation of microcirculation. HSCs play a key role in regulating hepatic microcirculation. In response to some stimuli, HSCs can contract or relax themselves, thus enlarging or shrinking the diameter of the sinusoidal lumen (36). Rho family of small GTPases regulates cell structure and motility mainly *via* rearrangement of actin cytoskeletons (37, 38). Rho-associated coiled-coil forming protein serine/threonine kinase (ROCK) was identified as one of the critical regulators of HSCs motility (39). In contrast to the scenario where HSCs appear to protect hepatocytes against IRI, HSCs drive liver injury mediated partly by ROCK. Administration of a specific inhibitor of ROCK named Y-27632 in rat attenuated IRI induced liver dysfunction manifested as increased deoxidized hemoglobin, decreased cytochrome oxidase, and elevated transaminase 1 h after reperfusion (40, 41). Consistently, Y-27632 resulted in relaxation of HSCs even in the presence of ET-1 *in vitro (* 41). Liver protective effect of ROCK inhibition on IRI was also confirmed by another study in rat with steatotic liver. Kuroda et al. demonstrated that suppressing ROCK with specific inhibitor fasudil ameliorated IRI induced increase in portal perfusion pressure and liver damage at early stage of IRI in steatotic liver (42). In particular, Rho/ROCK signaling in HSCs from steatotic livers was activated and the activation was related to increased contractility and ET-1, making it more vulnerable to IRI (42). Specificity of the effect of ROCK regulator on HSCs was further supported by the findings that HSCs targeting inhibition of ROCK by vitamin A-coupled liposomes suppressed HSC activation, hepatic blood supply, portal perfusion pressure during early hepatic IRI, and improved survival rate after the damage in rat steatotic liver (43).

A more clear landscape of functions of HSC in liver IRI is delineated by specific manipulating approaches for HSCs *in vivo*. Functional experiments in rodent model suggest that HSCs exacerbate injury during hepatic IRI mediated by TNF-α and ET-1. Exploiting genetically engineered mice expressing HSV-thymidine kinase under the GFAP promoter coupled with ganciclovir and CCL_4_ to eliminate actively proliferating HSCs, a seminal research by Stewart et al. showed that hepatic injury in both IRI and endotoxemia scenarios was attenuated in HSCs depleted mice (approximately 70%) (44, 45). The decreased injury was accompanied by significantly reduced hepatocyte pro-inflammatory cytokine TNF-α, neutrophil expression of chemoattractant CXCL1 and endothelin-A receptor (45). Of note, liver IRI and endotixin-induced acute injury might share similar cellular pathogenesis *via* HSC regulated inflammation. However, the time to evaluate the liver damage, mRNA and protein expression was not mentioned in this study, making it not feasible to infer whether HSCs regulate early or late liver IRI. Pharmacological approach alone has also been found to deplete HSCs *in vivo*. Gliotoxin induces apoptosis in both human and rat HSCs *in vitro*, and rat HSCs *in vivo* leading to resolution of fibrosis (46–48). Liver IRI in early phase was significantly reduced and sinusoidal perfusion was recovered by pretreatment with gliotoxin in HSCs decreased rat, suggesting HSCs exerted exacerbating effect on the magnitude of early liver ischemic injury (49).

Matrix metalloproteinases (MMPs) are a family of zinc-dependent proteases which are essential in the degradation of ECM to enable cellular movement and tissue reorganization (50). MMP activation and release are involved in liver IRI due to their profound effects on tissue integrity (51). It has been asserted that prolonged or over expression of MMP exerts harmful effects on the liver (52). Kupffer cells and HSCs are main sources of MMPs in the liver, although LSECs and leukocytes can also secrete MMPs (50, 53, 54). The involvement of MMPs in liver IRI is demonstrated by their concomitant expression and functional effects. HSCs contribute to MMP-9 production in the liver (55, 56). MMP-9 is increased 6 h after hepatic IRI in the steatotic rat orthotopic liver transplantation (OLT) model, and serum MMP-9 is elevated significantly 7 d post IRI in human OLT (57, 58). Furthermore, several MMPs, including MMP-9, are induced during early phase of liver IRI, and blocking MMPs with specific inhibitors reduces liver IRI and the release of proinflammatory cytokines (12, 59). MMP-9 deficiency and anti-MMP-9 neutralizing monoclonal antibodies also result in protection against damage during early phase of liver IRI in mice (60).

Despite the *in-vivo* data indicating HSCs exacerbate damage during IRI collectively, they may aid in or mediate liver protection *via* distinct signals. ROS have been proposed as key initiators of IRI response in the liver by causing direct cellular damage and inducing inflammatory response *via* high mobility group box‐1 (HMGB1) and NF-κB (61–63). An *in-vitro* study suggested that HSCs protected hepatocytes against ROS injury (64). In addition, pretreating mice with HSCs ameliorated liver IRI at 12 h after reperfusion in a regulatory T cells (Tregs)-dependent manner (65). It should be noted that, however, the HSCs administered were primary cell lines not subject to activation following hypoxia/reoxygenation (H/R) stress, which could largely explain the differences with HSC depletion results *in-vivo*. Post-conditioning with the volatile anesthetic drug sevoflurane protected the liver from IRI 1 d post reperfusion in a randomized controlled trial, and *in-vitro* study suggested that HSC might be the effector of the protection by reducing apoptosis of hepatocytes (66, 67). More specifically, supernatants of HSCs previously exposed to H/R induced apoptosis of hepatocytes, but this effect was attenuated with sevoflurane postconditioning (67). Fibroblast growth factor 10 (FGF10) belongs to the fibroblast growth factor (FGF) family, whose members play crucial roles in organ development, homeostasis, and repair (68). FGF10 binds to fibroblast growth factor receptor 2b (FGFR2b) and this signaling controls hepatoblast survival and liver size (69, 70). Li et al. demonstrated that HSCs secreted fibroblast growth factor 10 (FGF10) *in vitro*, which ameliorated inflammation and necrosis, and protected hepatocytes from apoptosis during early phase of liver IRI *in vivo (* 71). These results elucidated the protective effects of FGF10 in early liver IRI, and strongly implied these effects were modulated by HSCs.

## HSCs in liver repair and regeneration after IRI

The liver has a large regenerative capacity following physical or functional loss, with the potential of hepatocyte proliferation to sustain liver function. Necrotic tissue in the postischemic liver is cleared and remodeled by phagocytes, HSCs, and other cells, followed by hepatocyte regeneration and reconstruction of functional liver architecture (14). Far less is elucidated about the mechanisms of these processes compared with the mechanisms of hepatic IRI. Particularly, the role of HSCs in the process of liver repair after IRI is not clear (72).

MMPs derived from HSCs may promote liver repair after IRI in the liver, although they have been shown to promote damage by destruction of ECM and recruitment of leukocytes (73). Specifically, reduction in liver damage at 24 h after reperfusion and significant delay of liver repair after 72 h of reperfusion were observed in MMP-9 knockout mice, compared with wild type mice (74). MMP-9 was found to increase TGF-β activation after IRI. *In-vitro* study showed that MMP-9 activated TGF-β secreted by HSCs, indicating involvement of HSC in liver repair (74). A recent study exquisitely examined pathology of liver fibrosis during the repair process after IRI and highlighted involvement of HSC and MMPs. Konishi et al. found that the number of activated HSCs increased along the damaged areas 1 wk after IRI (72). Liver fibrosis took place at the interface between necrotic site and regenerating liver associated with HSCs during the reparative process after IRI, and noticeably, the number of HSCs decreased shortly after resolution of injury and restoration of disrupted liver structure. They also investigated the expression of several MMPs related to degradation of extracellular matrix components and reported that the expression of MMP-2 and MMP-9 increased at 1 wk after liver IRI and diminished thereafter. In contrast, MMP-13 expression remained at low level 1 wk after IRI but significantly elevated after 2 wk and the trend was stable up to 8 wk post IRI. The trends of MMP-2 and MMP-9 expression were associated with resolution of liver fibrosis and concomitant increase and decrease thereafter in the number of activated HSCs (72). Akin to MMP-9, MMP-13 is expressed in and produced by HSCs (75, 76). In relation to the injury stage, MMP-9 plays both deleterious and protective roles in hepatic IRI, which is dependent on the timing (74). It can be inferred from the pathological findings and source of MMPs that HSCs participate in the reparative process after liver IRI.

Recent innovative works involving Yes-associated protein (YAP) also underline the critical role of HSC in liver repair and regeneration after IRI. YAP and transcriptional coactivator with PDZ-binding motif (TAZ) are downstream effectors of the Hippo signalling pathway, which have been identified as essential regulators controlling cellular proliferation and organ size (77). Marked activation and proliferation of HSCs was observed at both injury and repair/regeneration phases after liver IRI in mice, with concurrent selective activation of YAP and TAZ and expression of their target genes. Inhibiting YAP and TAZ after injury phase significantly diminished HSC and hepatocyte proliferation, suggesting the dependence of liver repair and regeneration after IRI on HSC (78). Liu et al. demonstrated that YAP inhibition prior to ischemia and reperfusion operation delayed liver repair and increased hepatic fibrosis at 7 days after IRI. These changes were associated with enhanced HSC stimulation manifested as fibrogenic and contractile characteristics *in-vitro (* 79). This study indicated HSC might serve as a regulator of liver repair and fibrogenesis in an YAP dependent manner.

HSCs contribute to liver fibrosis during reparative process after IRI. ECM accumulation generated by HSCs potentiates at the boundary between necrotic and hepatocyte regenerating region. Resolution of liver fibrosis is associated with decreased activation of HSC (72). In fibrotic liver, HSCs may also promote liver repair after IRI. Fibrotic liver shows more severe injury but more rapid repair and regeneration compared with nonfibrotic liver in mice, which are accompanied by prominent accumulation of HSCs in fibrotic liver in early reparative phase (80).

## Intercellular communications involving HSCs in liver IRI

The signaling cascades leading to hepatic damage are various and complex, involving interactions between hepatocytes, Kupffer cells, HSCs, LSECs, recruited neutrophils, macrophages, and platelets (81). HSCs are highly versatile cells with complex crosstalk with residential hepatic cells and circulating immune cells, including hepatocytes, Kupffer cells, LSECs, natural killer cells (NK cells), T cells, and B cells (26, 27, 82, 83). This notion is demonstrated with enormous evidence mainly in the context of chronic hepatic injury leading to hepatic fibrosis, such as viral infection and alcoholic liver disease, but only a few works elucidate the crosstalk involving HSCs in liver IRI. As mentioned above, Kupffer cells are fundamental drivers of the early hepatic IRI. The crosstalk between Kupffer cells and HSCs were validated by *in-vitro* model (84, 85). This crosstalk was mediated by H_2_O_2_ and IL-6 (84). Furthermore, Kupffer cells can activate HSCs *in vitro* and *in vivo* mediated by IL-1 and TNF during fibrogenesis (86). It is believed that TNF-α and IL-6 released by Kupffer cells activate HSCs in the early phase of liver IRI (17). As liver fibrosis is a component of liver repair after IRI (72), Kupffer cells may induce activation and proliferation of HSCs in the recovery of IRI. In the scenario of liver fibrosis following chronic liver injury, activation and proliferation of HSCs are induced by TNF-α, IL-6, TGF-β, platelet-derived growth factor (PDGF), and ROS secreted by Kupffer cells (87, 88). CD4+ T cells are essential in promoting pro-inflammatory immune response in the liver and play an important role in hepatic IRI (89–91). Reifart et al. reported that CD4+ T cells interacted with HSCs along their migration to the liver *in vivo*. Depletion of HSCs diminished CD4+ T cell recruitment to the postischemic tissue and protected the liver from IRI (92). LSECs form the vascular wall of the liver sinusoid and play crucial protective roles in vascular homeostasis, and inflammation. LSECs are prominently vulverable to IRI, making them one of a key factors leading to hepatic IRI (81). LSECs suffering from ischemic challenge decrease production of NO, and together with elevated ET-1 production, contribute to contraction of HSCs. These events lead to narrowing of the sinusoidal lumen and microcirculatory dysfunction (81, 93). Cellular and molecular mechanisms by which HSCs regulate hepatic IRI are shown in **Figure 1**.

## Conclusion and future direction

Despite decades of research into the development of liver IRI and its intervention, liver IRI is still a major cause of mortality and morbidity after hepatic surgery and transplantation. Much less attention has been focused on the roles of HSCs in liver IRI compared to other cell types involved. HSCs become activated and proliferate in response to IRI, likely through signals from Kupffer cells. HSCs promote early phase hepatic IRI by constraining hepatic microcirculation mediated by ROCK, effects of ET-1 signalling, and pro-inflammatory cascades initiated by TNF-α. MMPs derived from HSCs may also increase damage by destruction of ECM and recruitment of leukocytes. HSCs can mediate hepatic protective effect *via* external stimuli such as sevoflurane, and FGF10. During the repair and regeneration stage, HSCs play an fundamental role in potentiating liver recovery. Molecular mechanisms involve activation of TGF-β signalling pathway by MMP-9, activation of YAP and TAZ. During the reparative stage of liver IRI, HSCs also regulate fibrogenesis, the extent of which may be critical to functional recovery of the liver.

Future research regarding involvement of HSCs in liver IRI can be aimed at three directions to aid in better understanding of the pathophysiology of IRI and development of novel therapeutic interventions. Intercellular communications between HSCs and other cell types should be studied using *in vivo* visualization techniques and cell-type specific genetic animal models. Furthermore, it is clinically useful to identify HSCs derived biomarkers predictive of transplantation outcomes with less expensive modern multi-omics technologies. It is of paramount importance to screen and identify novel agents to ameliorate hepatic IRI, given that clinical trials of many drugs targeting HSCs for anti-fibrosis are completed or under way (26). An update of the clinical trials and drugs is shown in **Table 1** (94–99). Because HSCs contribute to damage and repair of liver IRI, it is likely that anti-fibrotic drug has an effect on combating IRI.

**Table 1:** Clinical trials of drugs targeting HSC activation.

Drug	Target	Main findings	Phase of Trial	Status	NCT number	Reference
Pioglitazone	PPARγ agonist	Reduces serum aminotransferase levels, hepatic steatosis, lobular inflammation for NASH without diabetes	III	completed	NCT00063622	(94)
Obeticholic acid	Farnesoid X receptor agonist	Increases insulin sensitivity, reduces biomarkers of liver inflammation and fibrosis for NFAD with T2DM	II	completed	NCT00501592	(95)
		Improves liver fibrosis and NASH disease activity for NASH	III	active	NCT02548351	(96)
Elafibranor	Dual PPARα– PPARδ ligand	Induces resolution of NASH without worsening fibrosis in patients with NASH without cirrhosis	III	completed	NCT01694849	(97)
Cenicriviroc	Dual CCR2– CCR5 receptor antagonist	Improves liver fibrosis without impacting steatohepatitis for NASH with liver fibrosis	II	completed	NCT02217475	(98)
Belapectin(GR-MD-02)	Galectin-3 antagonist	Does not improve liver fibrosis for NASH with liver fibrosis evaluated by imaging methods	II	completed	NCT02421094	N/A
		Does not improve portal hypertension in patients with NASH, cirrhosis, and portal hypertension, but reduces portal pressure in patients without esophageal varices and varices development	II	completed	NCT02462967	(99)
ND-L02- s0201	HSP47	N/A	I	completed	NCT02227459	N/A

NASH, nonalcoholic steatohepatitis; NFAD, nonalcoholic fatty liver disease; T2DM, type 2 diabetes mellitus; N/A, not applicable.

## Author contributions

YP, MY, LC, XS, WX, JL, and QY contributed to the writing and editing of the manuscript. All authors contributed to the article and approved the submitted version.

## Funding

This study was supported by Technical Innovation Project of Hunan Provincial Health Commission (No.: Memo [2018]187 of Xiangwei Medical Administration Office), International Talent Project of Hunan Children’s Hospital.

## Conflict of interest

The authors declare that the research was conducted in the absence of any commercial or financial relationships that could be construed as a potential conflict of interest.

## Publisher’s note

All claims expressed in this article are solely those of the authors and do not necessarily represent those of their affiliated organizations, or those of the publisher, the editors and the reviewers. Any product that may be evaluated in this article, or claim that may be made by its manufacturer, is not guaranteed or endorsed by the publisher.
